# Efficient transplacental transfer of SARS-CoV-2 antibodies between naturally exposed mothers and infants in Accra, Ghana

**DOI:** 10.1038/s41598-024-61496-3

**Published:** 2024-05-10

**Authors:** Frederica D. Partey, Dorotheah Obiri, Evelyn Yayra Bonney, Abigail Naa Adjorkor Pobee, Isaac Kumi Damptey, Keren Ennuson, Jayln Akwetea-Foli, Franklin Yengdem Nuokpem, David Courtin, Kwadwo A. Kusi, Benedicta A. Mensah

**Affiliations:** 1 Department of Epidemiology, Noguchi Memorial Institute for Medical Research, College of Health Sciences, University of Ghana, P.O BOX LG 581, Legon, Accra, Ghana; 2 Department of Biochemistry, Cell and Molecular Biology, University of Ghana, Accra, Ghana; 3 Université Paris Cité, IRD, MERIT, 75006 Paris, France

**Keywords:** COVID-19, SARS-COV-2, Maternal COVID-19, Prenatal COVID-19 placental transfer, Transfer efficiency, Infectious diseases, Immunology, Infectious diseases, Viral infection

## Abstract

We aimed to determine SARS-CoV-2 antibody seropositivity among pregnant women and the transplacental transfer efficiency of SARS-CoV-2-specific antibodies relative to malaria antibodies among SARS-CoV-2 seropositive mother-cord pairs. This cross-sectional study was conducted in Accra, Ghana, from March to May 2022. Antigen- specific IgG antibodies against SARS-CoV-2 (nucleoprotein and spike-receptor binding domain) and malarial antigens (circumsporozoite protein and merozoite surface protein 3) in maternal and cord plasma were measured by ELISA. Plasma from both vaccinated and unvaccinated pregnant women were tested for neutralizing antibodies using commercial kit. Of the unvaccinated pregnant women tested, 58.12% at antenatal clinics and 55.56% at the delivery wards were seropositive for both SARS-CoV-2 nucleoprotein and RBD antibodies. Anti-SARS-CoV-2 antibodies in cord samples correlated with maternal antibody levels (N antigen r_s_ = 0.7155, *p* < 0.001; RBD r_s_ = 0.8693, *p* < 0.001). Transplacental transfer of SARS-CoV-2 nucleoprotein antibodies was comparable to circumsporozoite protein antibodies (*p* = 0.9999) but both were higher than transfer rates of merozoite surface protein 3 antibodies (*p* < 0.001). SARS-CoV-2 IgG seropositivity among pregnant women in Accra is high with a boost of SARS-CoV-2 RBD-specific IgG in vaccinated women. Transplacental transfer of anti-SARS-CoV-2 and malarial antibodies was efficient, supporting vaccination of mothers as a strategy to protect infants against SARS-CoV-2.

## Introduction

In May 2023, the World health organisation (WHO) declared coronavirus disease (COVID-19) pandemic which began in 2019 over. As at the time of declaration, there were an estimated 765 million confirmed cases of COVID-19 globally, with Africa accounting for less than 2% of the confirmed COVID-19 cases^1^. COVID-19-related morbidity and mortality in sub-Saharan Africa is comparatively lower than that in developed countries^2^. Significantly low testing rates in many African countries due to limited technical capacity and scarce resources mean that the true disease burden of COVID-19 is underestimated^3^. In addition, reduced testing leads to the challenge of identifying at-risk populations within each local context and targeting vulnerable populations for interventions.

SARS-CoV-2 infection during pregnancy, particularly in the third trimester, increases the risk of severe disease and results in poor obstetric outcomes^4–6^. Due to the vulnerability of pregnant women, many countries have prioritized pregnant women for interventions such as vaccination. Over 60% of global pregnancies occur in resource-limited settings, where the rates of maternal mortality and adverse pregnancy outcomes are disproportionately high in relation to developed parts of the world^7^. It is therefore critical to determine the burden of SARS-CoV-2 exposure among the pregnant population in Africa to inform population-based policy decisions in order to mitigate the impact of COVID-19 and any future disease threats on maternal and neonatal health. Several seroepidemiological studies from different African countries have reported significantly high SARS-CoV-2 seropositivity rates within a wider population, which confirms the underestimation of COVID-19 cases in Africa based on reported testing figures^8–10^. It is possible COVID-19 burden among the pregnant population in Africa might diverge from estimates in most developed countries. There are a few studies examining the prevalence of SARS-CoV-2 infection among African pregnant women^10–15^ which point to an increase in SARS-CoV-2 seropositive rates over the course of the pandemic. However, only one study examined effects of prenatal COVID-19 exposure on birth outcomes^15^ and transplacental efficiency of SARS-CoV-2 IgG alone but did not compare SARS-CoV2 transfer efficiency to other antigen-specific IgG.

Aside COVID-19 pandemic, sub-Saharan Africa remains endemic to other infectious diseases, such as malaria, tuberculosis, and HIV. Over 90% of global malaria-associated morbidity and mortality occur in sub-Saharan Africa, with pregnant women and children under five years having the greatest risk of severe disease. However, in such endemic populations, infants 0–6 months rarely develop malaria and are resistant to severe disease, a phenomenon attributed to the placental transfer of malaria-specific immunoglobulins (Igs) from mothers to fetuses^16^. The presence of infections can affect the efficiency of maternofetal transfer of IgG from mothers to newborns, as has been shown in HIV-infected pregnant women^17^. Studies conducted in developed countries have shown reduced placental transfer of SARS-CoV-2-specific IgG from mothers to infants^18,19^. In sub-Saharan Africa where infectious disease burden is high, it is therefore critical to understand how COVID-19 infection in pregnancy affects the efficiency of placental transfer of IgG against common infections such as malaria.

In the present study, we determined SARS-CoV-2 antibody seropositivity among pregnant women attending routine antenatal clinics and parturient women admitted to delivery wards. We further examined the transplacental transfer efficiency of SARS-CoV-2-specific antibodies in relation to the transfer of malaria-specific antibodies among mother-infant dyads.

## Results

### Participant characteristics

Pregnant women were recruited from Maamobi General Hospital in Accra, Ghana. Participants’ age ranged from 15 to 47 years (Table 1). Over 80% of the study population had at least some basic education with most of them employed in the informal sector. Few (5%) of our participants had existing comorbidities. Only 9.2%(55/599) of the participants had received at least a single dose of COVID-19 vaccines. All Naso- and oropharyngeal swabs from the pregnant women tested in RT-PCR were negative for SARS-CoV-2 indicating none of the pregnant women attending ANC or admitted to the delivery wards had an active SARS-CoV-2 infection during the study period.

**Table 1:** Maternal characteristics at enrolment.

	Total	Pregnant women at ANC	Pregnant women at Delivery
	N = 599	N = 400	N = 199
		N (%)	N (%)
Age Groups (years) Median (range)	29 (15–47)		
< 25	164 (27.38)	110 (27.5)	54 (27.14)
26–30	188 (31.39)	124 (31)	64 (32.16)
30–35	114 (19.03)	72 (18)	42 (21.1)
> 35	133 (22.2)	94 (23.5)	39 (19.60)
Education Level			
None	55 (9.18)	44 (8.0)	11 (5.53)
Basic	290 (48.41)	193 (10.3)	97 (48.74)
Secondary	203 (33.89)	128 (33.2)	75 (37.69)
Tertiary	51 (8.51)	35 (9.0)	16 (8.04)
Occupation			
Unemployed	102 (17.03)	64 (16)	38 (19.1)
Informal	451 (75.29)	309 (77.25)	142 (71.36)
Formal	46 (7.68)	27 (6.75)	19 (9.55)
Gestation Age			
0-14wks	18 (3)	18 (4.5)	–
14 + 1–28 wks	155 (25.87)	155 (38.75)	–
28 + 1-40wks	426 (71.12)	227 (55.25)	199 (100)
Comorbitidies			
High blood pressure	21 (3.51)	17 (4.25)	4 (2)
Diabetes	3 (0.5)	2 (0.5)	1 (0.5)
Asthma	5 (0.83)	3 (1.5)	2 (1)
Kidney-related diseases	1 (0.17)	1 (0.28)	–
Received at least a single dose of COVID-19 vaccines	55 (9.19%)	46 (12.99)	9 (4.5)

### SARS-CoV-2 IgG prevalence

**Figure 1:**
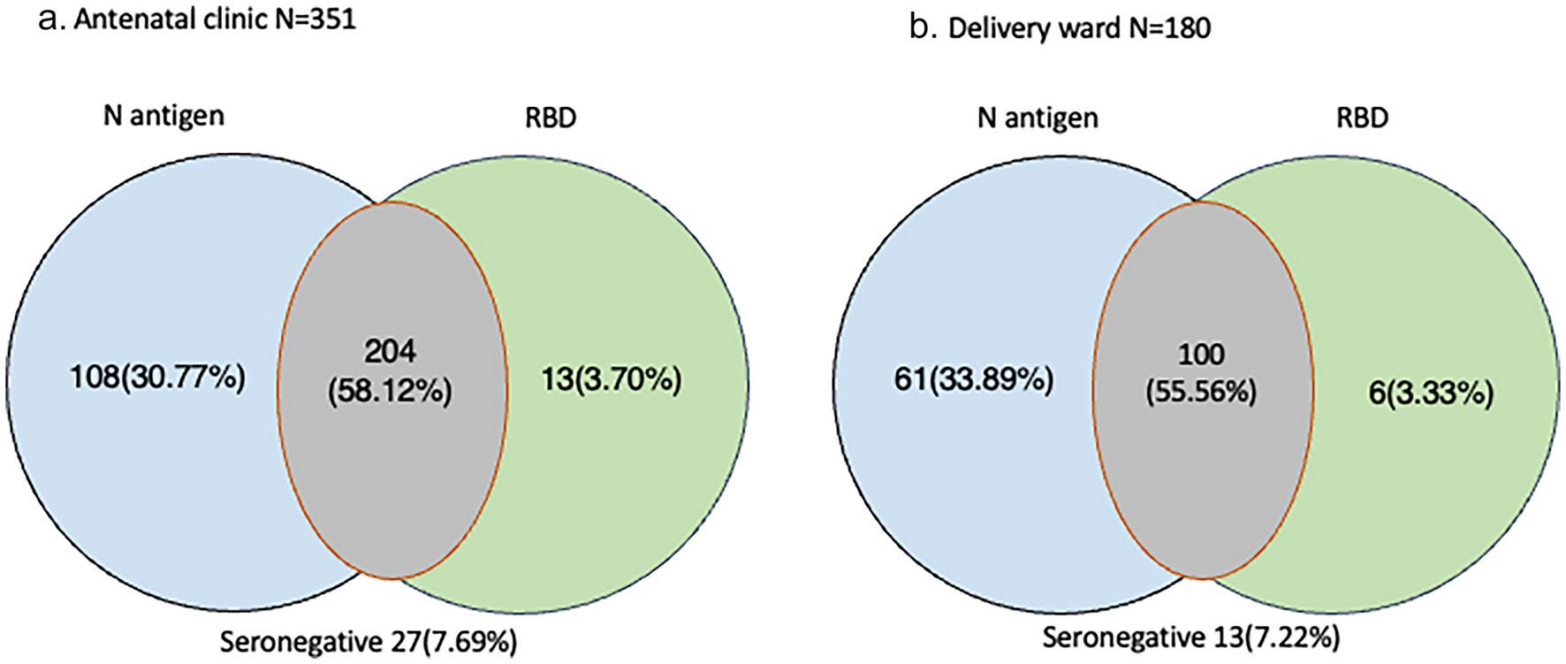
Seroprevalence to SARS-CoV-2 is high in pregnant women. IgG antibody seropositivity of SARS-COV-2 N-antigen (**a**) and RBD antigen (**b**) in plasma from pregnant women at antenatal and delivery wards. Seropositivity against N antigen alone (blue), RBD alone (green) and both antigens (grey).

At the time of conducting the study, vaccination was recommended for pregnant women in Ghana. In examining SARS-CoV-2 antibody seropositivity, individuals who had received at least one dose of COVID-19 vaccines were excluded from this analysis (Fig. 1). Of the 351 women at the antenatal clinics who were unvaccinated, 204 (58.12%, 95% CI 52.75–63.30%) were seropositive for both SARS-CoV-2 N antigen and RBD, 108 (30.77%, 95% CI 26.04–35.93%) and 13 (3.7%, 95% CI 2.07–6.41%) showed IgG reactivity to only N antigen and RBD respectively (Fig. 1a). The remaining 27 (7.69%, 95% CI 5.22–11.12) women were seronegative against both antigens. A similar patten was observed among the parturient women at the delivery wards (Fig. 1b). Out of the 180 women who were unvaccinated and were tested, 100 (55.56%, 95% CI 47.98–62.89) elicited a positive IgG response against both N antigen and RBD, 61 (33.89%, 95% CI 27.12–41.36) were IgG positive for N antigen alone and 6 (3.33, 95% CI 1.36–7.45) were IgG positive for RBD alone. In all, 13 (7.22%, 95% CI 4.06–12.30) women at the delivery wards did not have antibodies against neither N antigen or RBD.

### Transplacental transfer efficiency of SARS-CoV-2 antibodies

**Figure 2:**
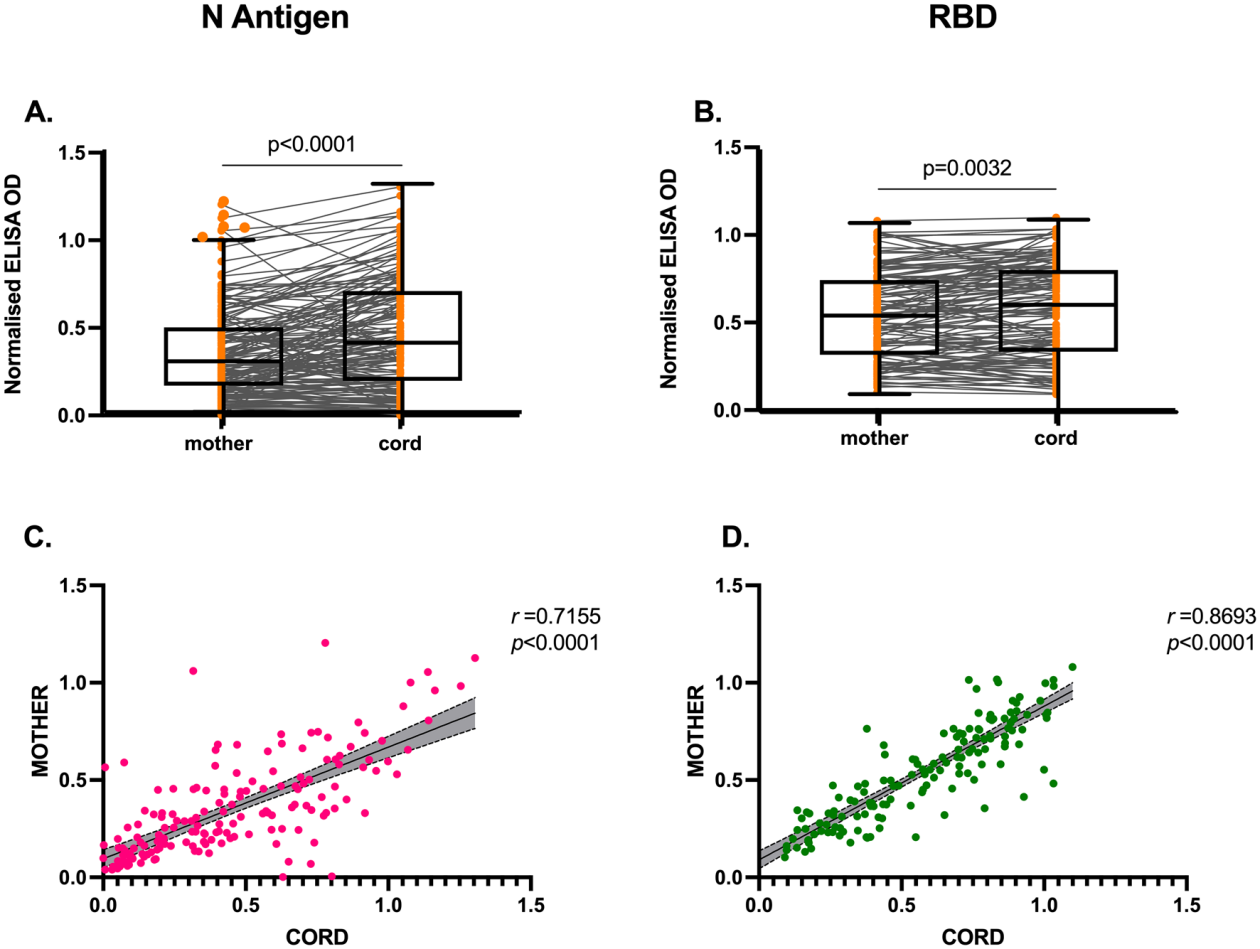
Placental transfer of IgG between seropositive mothers to matching cord. SARS-CoV-2 antibodies against nucleoprotein (N) protein (**A**) and RBD (**B**) between mother:cord pairs. Spaghetti lines represent antibody levels between mother and matching cord sample. Box and whisker represent median with interquartile range. Correlation between maternal and cord IgG antibody levels against nucleoprotein (**C**) and RBD (**D**) presented in linear regression. Shaded are depicts 95% confidence interval. *r* represents the spearman’s rank coefficient .

We next determined the transfer efficiency of SARS-CoV-2 antibodies between mothers who were seropositive (n = 170) and their matching cord samples (Fig. 2). SARS-CoV-2 N antigen and RBD-specific IgG antibody levels in cord plasma was significantly high than in mothers (*p* < 0.0001, Fig. 2A; *p* < 0.0032, Fig. 2B). We found positive correlations between seropositive mother plasma antigen-specific antibody levels and corresponding cord sample for both N antigen (*r* = 0.7155, *p* < 0.0001, Fig. 2C) and RBD (*r* = 0.8693, *p* < 0.0001, Fig. 2D).

**Figure 3:**
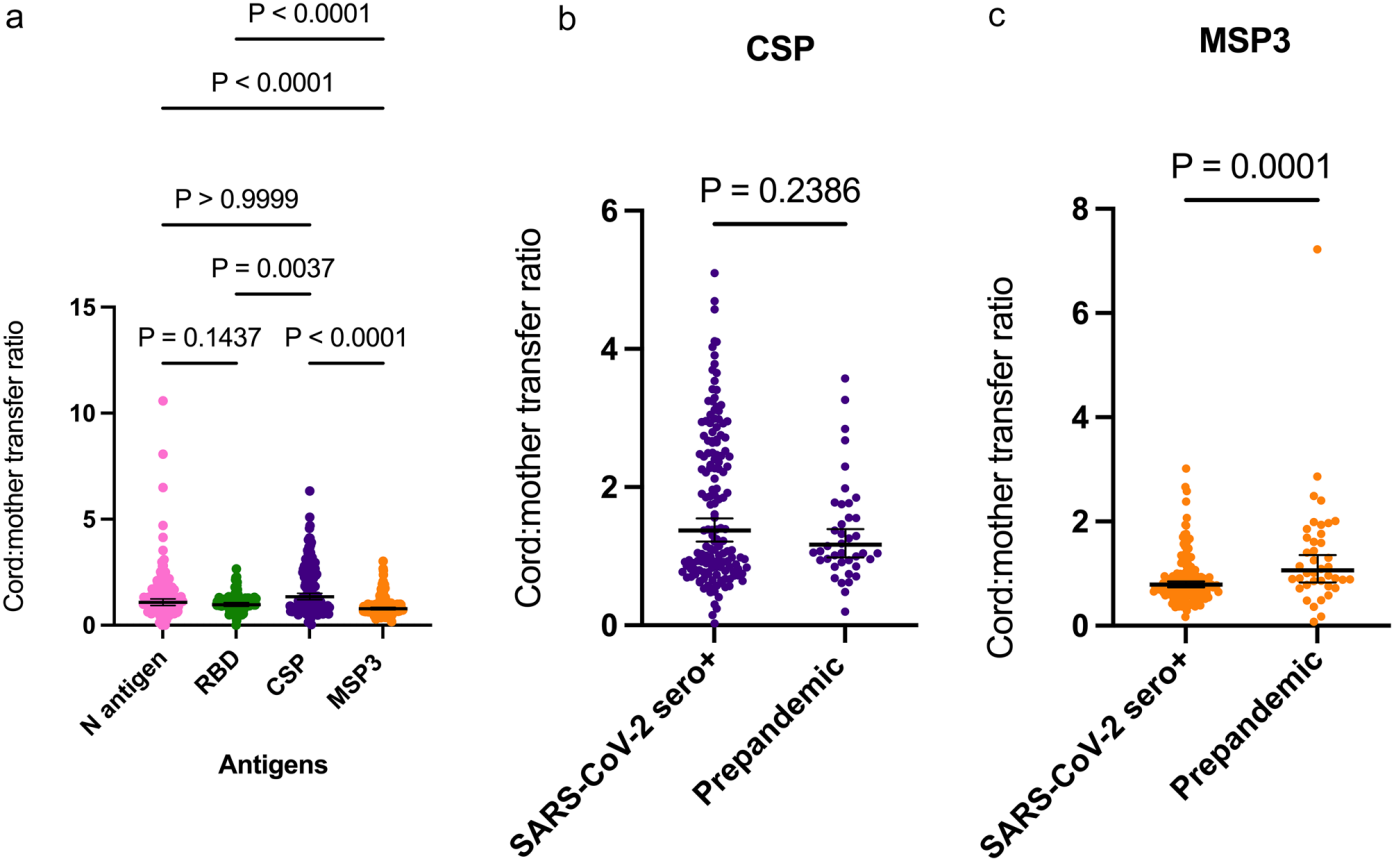
Transfer efficiency of SARS-CoV-2 and malaria-specific antibodies. Cord:maternal antibody transfer ratio of antigens among SARS-CoV-2 seropositive individuals (**a**). Transfer ratio of CSP (**b**) and MSP3 (**c**) among SARS-CoV-2 seropositive paired mother:cord plasma samples and pre-pandemic paired mother:cord plasma samples. Transfer ratio was calculated as: (cord IgG concentration)/(maternal IgG concentration). Kruskal Wallis test was used to analyse comparisons in A. Mann–Whitney test was used to compare transfer ratios in b and c. Data is presented as geometric mean (thick horizontal lines) and 95% confidence interval (error bars).

As a comparator for transplacental efficiency, we estimated the cord:mother transfer ratio of antibodies against malaria pre-erythrocytic stage antigen CSP and merozoite antigen MSP3 among SARS-CoV-2 seropositive mothers and paired cord samples (Fig. 3a).There was no difference in the mean transfer ratio between N antibodies (mean = 1.08, 95% CI 0.94–1.24) and CSP antibodies (mean = 1.35, 95% CI 1.20–1.51). Both N antigen and CSP antibody transfer ratio was significantly high than RBD (mean = 0.89, 95% CI 0.89–1.06) and MSP3 antibody transfer ratio (mean = 0.733, 95% CI 0.73–0.84) with MSP3 being the least transferred. We subsequently compared the placental transfer ratios of CSP and MSP3 antibodies between SARS-CoV-2 seropositive mother:cord pairs and paired mother:cord plasma samples collected between 2016–2017 before the onset of COVID-19 pandemic. There was no difference in the cord:mother transfer ratios of CSP antibodies between SARS-CoV-2 seropositive individuals and the prepandemic samples (Fig. 3b). In contrast, MSP3-specific cord:mother transfer ratios were significantly high in the prepandemic samples than the SARS-CoV-2 seropositive individuals (Fig. 3c). Generally, CSP transfer ratio was high compared to MSP3.

### Transfer of SARS-CoV-2 neutralizing antibodies

**Figure 4:**
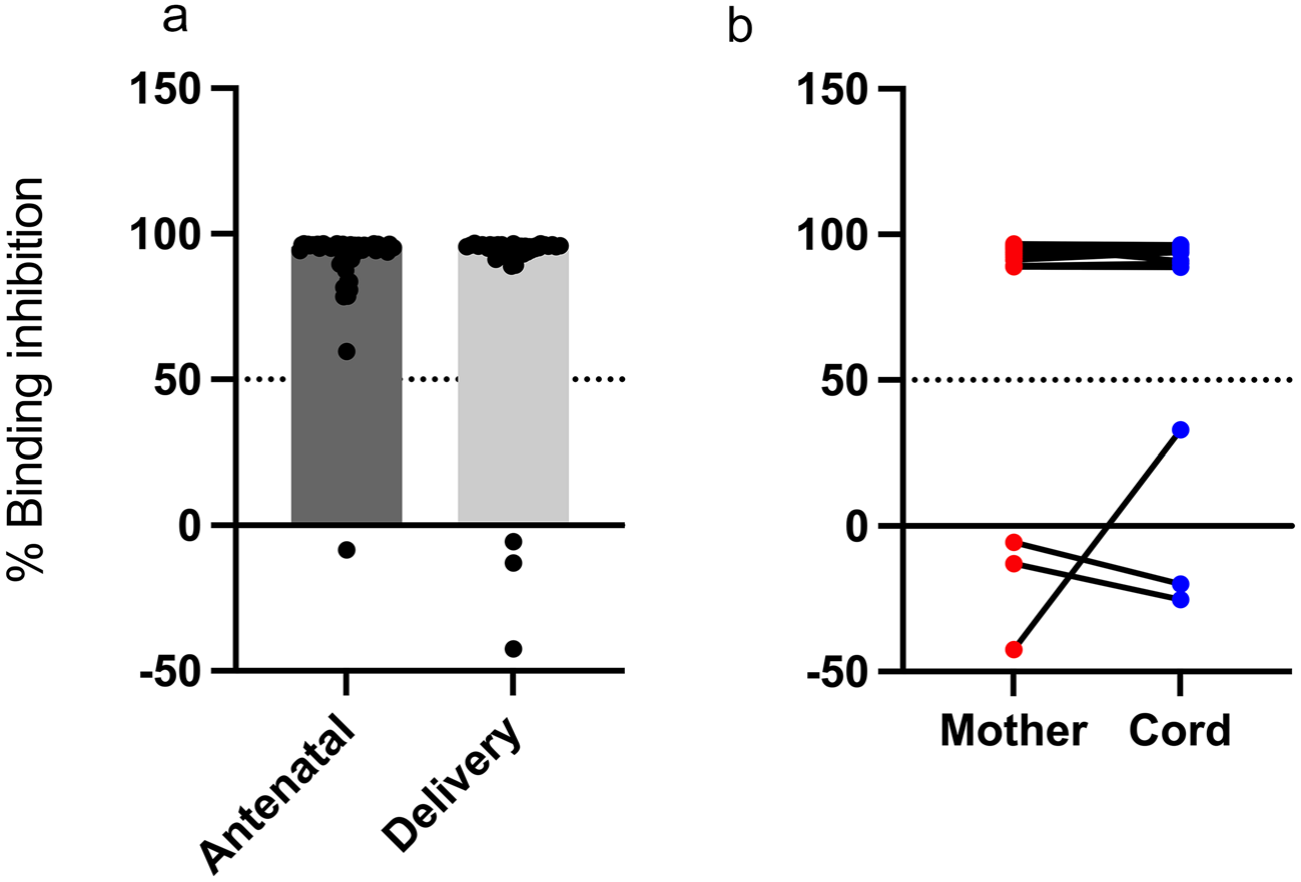
Inhibition of ACE2 binding in SARS-CoV-2 seropositive pregnant. Percentage binding inhibition between seropositive pregnant women at antenatal clinics and at delivery (**a**). Inhibitory antibodies transferred from mothers to cord (**b**). Bars indicate median binding inhibition. Horizontal dashed lines represent 50% inhibition.

To examine the functionality of SARS-CoV-2 antibodies induced in individuals, we used plasma from a subset of the pregnant women who were seropositive for SARS-CoV-2 in a competitive ELISA (Fig. 4). Significantly high proportion of the seropositive pregnant women both at ANC (60/69) and delivery wards (n = 41/44) had neutralizing antibodies (Fig. 4a). Among the parturient women tested, the neutralizing antibodies were effectively transferred to cord (Fig. 4b) except between mothers who were seronegative for neutralizing antibodies and their matching cord.

### Vaccination boost spike- RBD antibodies

**Figure 5:**
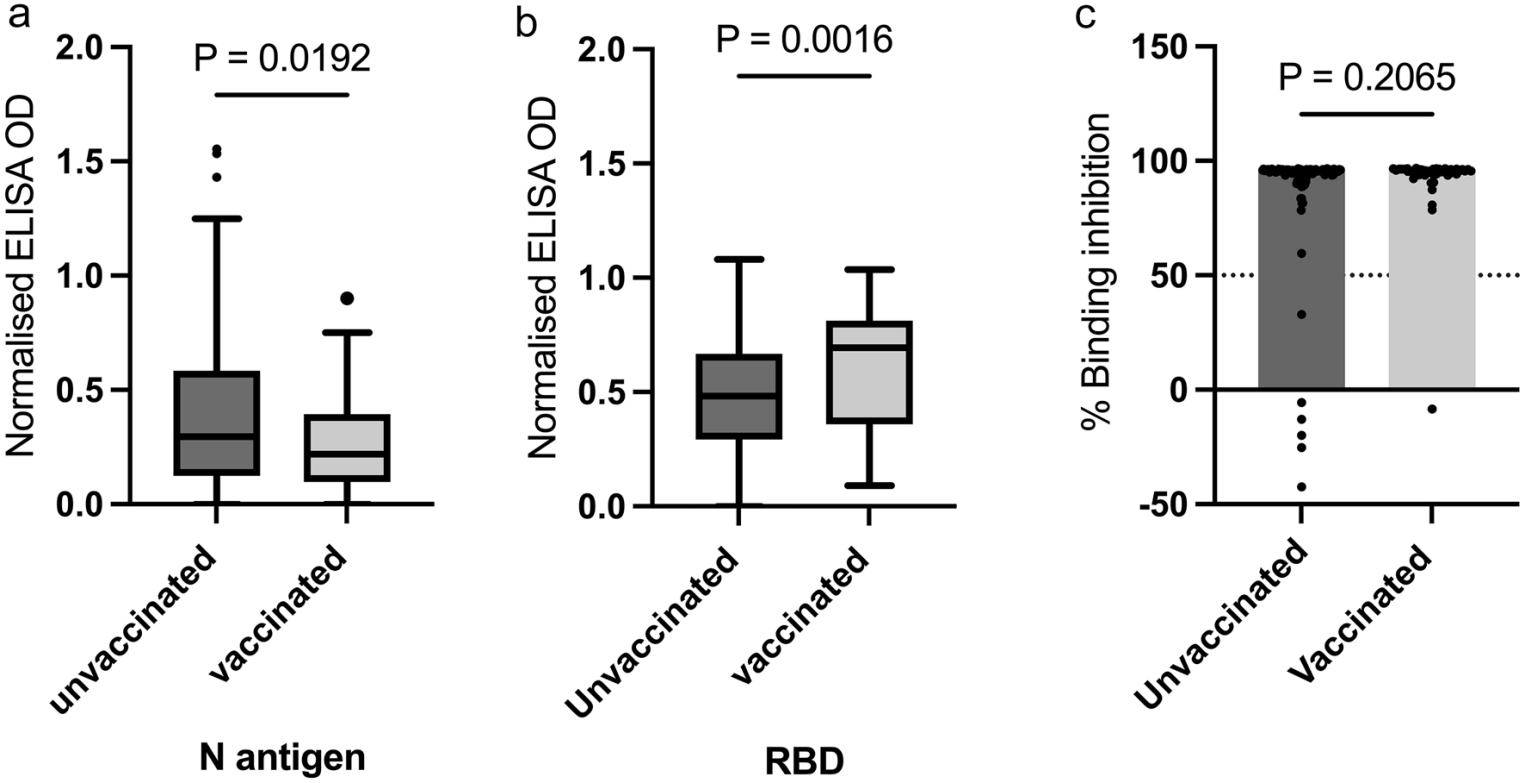
Vaccination against SARS-CoV-2 causes a boost in spike-RBD specific antibodies. Antibody levels against N antigen (**a**) and Spike-RBD (**b**) between pregnant women vaccinated against COVID-19 and unvaccinated pregnant women. ACE-2 binding inhibition rates between pregnant women vaccinated against COVID-19 and unvaccinated pregnant women (**c**). Mann–Whitney test was used to analyse comparisons between groups.

Our results show that a significantly high proportion of the participants had been naturally exposed to SARS-CoV-2 over the course of pandemic. Thus, we compared SARS-CoV-2 antibody levels between pregnant women naturally exposed to COVID-19 who were unvaccinated and naturally exposed women who had received at least a single dose of COVID-19 vaccine before or during pregnancy (Fig. 5). Median plasma N-antigen IgG levels were significantly increased among women who were unvaccinated compared to vaccinated women (p = 0.0192, Fig. 5a). Conversely, anti-RBD IgG levels were significantly boosted in the vaccinated women relative to the unvaccinated naturally exposed women (*p* = 0.0016, Fig. 5a). However, the percentage inhibition of ACE-2 binding was comparable (*p* = 0.2065) between vaccinated (median = 95.53%) and unvaccinated pregnant women (95.51%).

## Discussion

Pregnancy predisposes pregnant women to severe disease outcomes when infected with SARS-CoV-2 compared to non-pregnant individuals. In Africa, COVID-19 testing capacities during the pandemic was limited in many countries particularly in sub-Saharan Africa. Levels of SARS-CoV-2 exposure among pregnant women in sub-Saharan is not fully known as data from such serosurveys is important in designing interventions targeting the vulnerable pregnant population to current and future disease threats. Our key findings suggest SARS-CoV-2 antibody prevalence among pregnant women in urban Accra is high (55–58%). COVID-19 vaccination rate was markedly low among our participants. We found that SARS-CoV-2 RBD-specifc IgG is significantly boosted in vaccinated women relative to unvaccinated women. SARS-CoV-2 specific antibodies are efficiently transferred between mother and neonates as there was strong correlation between mother and cord samples. The transplacental transfer rate of SARS-CoV-2 antibodies was comparable to malaria-specific antibodies among SARS-CoV-2 seropositive individuals.

Previous studies examining SARS-CoV-2 seropositivity among HIV-infected pregnant women in Mozambique revealed a 11.3% seroprevalence in 2021^14^. Similar studies conducted in Ethiopia recorded a prevalence of 5.7% in pregnant women between April 2020 and March 2021^20^. In Accra, Ghana, nucleoprotein seropositivity rates among the general non-pregnant population was found to have increased from 19.8% in November, 2020 to 42.7% in July, 2021^9^. Our seroprevalence data is much higher than the seroconversion rates recorded in the earlier study in Ghana and other African countries. At the time of conducting the present study, Ghana had experienced 4 major waves^21^. The high seroprevalence rates we observed is suggestive of ongoing transmission which increased with each subsequent wave as demonstrated in a study conducted among pregnant women in Gambia between 2020 and 2021^10^. Although majority of the seropositive individuals had detectable antibodies against both the nucleoprotein and RBD antigens, some individuals showed seropositivity against only one antigen. Similar observations have been made in earlier serosurveys among the wider non-pregnant population in Ghana^9^ and other LMICs^22^. It is thought that in some individuals, there is preferential seroconversion of antibodies against one antigen over the other. This was reported in comparative antibody profile studies in children and adults where children were observed to have mainly anti-spike antibodies but not anti-nucleoprotein antibodies^23^.

Based on the physical examination and interviews, none of our study participants were symptomatic and seropositivity was not associated with any adverse birth outcomes. Studies in other populations in both developed and resource-limited settings have shown that PCR-confirmed COVID-19 diagnosis in pregnant women increased risk of adverse birth outcomes compared to undiagnosed pregnant women^5,24,25^.

Transplacental SARS-CoV-2 RBD IgG transfer was found to be impaired in symptomatic pregnant women relative to asymptomatic pregnant women^18,19,26^. In the present study, we observed efficient transfer of SARS-CoV-2 nucleoprotein and RBD antibodies from mothers to neonates. Our results are in agreement with similar studies which found passive transfer of SARS-CoV-2 antibodies between PCR-positive pregnant women who were asymptomatic^27^. Since all our participants are asymptomatic, it is plausible some women were infected early on in their pregnancies allowing enough time for mothers to develop antibodies which are transferred to neonates. Antibodies in cord samples strongly correlated with maternal antibody levels in line with studies both in natural infections and vaccination^27–29^. Transplacental rates of malaria-specific antibodies (anti-CSP and MSP3) were similar between SARS-CoV-2 seropositive mother:cord pairs and pre-pandemic paired mother:cord samples in contrast to reports by others who found reduced transfer rates of influenza and diphtheria antibodies in COVID-19 infected pregnancies compared to uninfected pregnancies^19^. These observations are reassuring considering that malaria remains an important public health disease in sub-Saharan Africa and the passive transfer of malaria-specific antibodies are important in immunity against malaria in the first six months of life.

At the time of conducting the study, COVID-19 vaccination was recommended for pregnant women by the Ghana Health Service. Despite our participants knowledge about the importance of vaccination in protection against COVID-19^30^, vaccine uptake was significantly low. Vaccination significantly increased SARS-CoV-2 RBD antibodies in the vaccinated women compared to natural infections. Our finding is unsurprising given that the COVID-19 vaccines target the spike RBD. Regardless of the marked increase in the RBD levels, we did not find any difference in the levels of neutralizing antibody between the vaccinated and unvaccinated pregnant women. From the vaccination records, majority of the respondents had received only a single dose of the viral-vectored Oxford-AstraZeneca vaccine and these vaccinations for most of the women were before the current pregnancies. Thus, the number of doses and timing of these immunizations may impact the durability and function of vaccine-induced antibody responses.

Our study had some limitations as discussed below: First, the cross sectional study design did not allow us to follow pregnant women and infants up to determine durability of SARS-CoV-2 specific antibodies. Prior work has shown that vaccination in pregnant women induce more durable SARS-CoV-2 protective antibodies compared to infection-induced antibodies^31^. Secondly, in comparing the neutralizing antibodies for vaccinated and non-vaccinated pregnant women, we used a kit that has recently been shown to have reduced sensitivity to emerging variants particularly post-omicron era^22,32^. Molecular surveillance data from Ghana published after the present study was conducted showed that the predominant circulating strain during the study period was the omicron variant^21^. This may explain why the elevated anti-RBD antibodies seen in vaccinated mothers versus unvaccinated mothers did not translate to an increased percent of inhibition of ACE-2 binding in our study.

Thirdly, the absence of RT-PCR positive cases in our study population limited our ability to investigate fully, the effects of active prenatal COVID-19 on birth outcomes within our population. Additionally, due to the low vaccination rates among our participants, we were unable to compare transfer efficiencies between COVID-19 vaccinated and unvaccinated individuals. However, the efficient transfer of naturally acquired antibodies from mothers to neonates together with the strong correlation between maternal and infant antibody levels in our population supports the immunisation of pregnant women to ensure optimal protection of infants during the first few weeks of life.

In conclusion, we have shown higher SARS-CoV-2 antibody seropositivity in pregnant women in Accra. There is efficient transplacental transfer of SARS-CoV-2 antibodies comparable to malaria-specific antibodies in SARS-CoV-2 seropositive pregnancies. The study highlights the importance of maternal immunisation to enhance neonatal health.

## Methods

### Study participants

The study was a cross sectional prospective study conducted from March to May 2022 at the Maamobi General Hospital, Accra, Ghana. Pregnant women attending routine antenatal clinics (ANC) and parturient women admitted to the delivery wards were enrolled in the study. Pregnant women at ANC were sampled during the month of March 2022 while samples from parturient women were collected from March to May 2022. Naso- and oropharyngeal swabs were collected from all the pregnant women into virus transport media. Heparinized venous blood was drawn from participants at both ANC and delivery wards. At the delivery wards, matching cord blood was drawn immediately after clamping the cord. Plasma was separated by centrifugation and stored at -20°C until use. A structured questionnaire was administered to study participants to capture socio-demographic, clinical and vaccination status. In addition, participants were screened by the medical team for fever, cold, cough, loss of smell or taste, chest tightness and breathing difficulty within 48 h before enrolment. Anthropometric data including gestational age, birth weight, length of baby and head circumference were recorded after delivery and was used in assessing birth outcomes.

The study was approved by the Institutional Review Board of Noguchi Memorial Institute for Medical Research (NMIMR-IRB CPN 047/21-22) and the Ghana Health Service Ethics Review Committee (GHS-ERC 021/11/22). Participation in the study was voluntary and informed consent was obtained from all volunteers before taking part in the study. All methods were carried out in accordance with relevant guidelines and regulations.

### RT-PCR for SARS-CoV-2 detection

Naso- and oropharyngeal swabs in virus transport medium were transported to the Virology lab at Noguchi Memorial Institute for Medical Research for RT-PCR detection of SARS-CoV-2. Viral ribonucleic acid (RNA) was extracted using the RADI PREP DNA/RNA kit (KH Medical) following the manufacturer's protocol. The extracted RNA was tested in RT-PCR using Veri-Q Prep M16 system (MiCoBiomed, Korea) with primer sets and probes targeting the open reading frame (ORF3a), envelope genes (E) and SARS-CoV-2nucleoprotein following the manufacturer’s protocol and has been described in detail previously^33^. Cycle threshold less than 40 was considered positive for SARS-CoV-2. All RT-PCR was run within 24 h after sample collection.

### Protein expression

The SARS-CoV-2 nucleoprotein (N) was was expressed as detailed in^34^. Briefly, the protein was expressed from the plasmid pHYRSF53 (Addgene #64696), containing the SARS-CoV-2 nucleocapsid gene (NCBI Gene ID: 43740575) fused with a cleavable SUMO-His-tag nucleotide sequence in Escherichia coli BL21 (DE3) cells. After Ni-NTA purification, the SUMO-His-tag was cleaved and further purified using Ni-NTA beads to obtain a tag-free nucleocapsid antigen. The receptor binding domain (RBD) of the spike protein of the SARS-CoV-2 ancestral strain was expressed in 293 freestyle cell systems as described elsewhere^35^. *Plasmodium falciparum* circumsporozoite protein (CSP) and merozoite surface protein (MSP3) of the 3D7 clone were expressed in the *Lactococcus lactis* system and as described elsewhere in detail^36^.

### Antigen-specific IgG reactivity

To measure antigen-specific IgG in plasma, an indirect ELISA was used as described previously^37^ with a few modifications. In summary, Nunc Maxisorp plates were coated with antigens at 0.5 µg/mL (N antigen), 0.5 µg/mL (RBD antigen), 1 µg/mL (CSP), 0.5 µg/mL (MSP3) and stored at 4°C overnight. Plates were washed with PBS/tween and blocked with BSA/PBS-Tween for 1 h. Following blocking, plasma was added to wells at a dilution of 1:100 for all antigens except MSP3 which was diluted at 1:200. Plasma pools from COVID-19 convalescent individuals and archived pre-pandemic samples from non-pregnant adults were used as positive and negative plasma control respectively to normalize plate-plate variation. After plasma incubation, plates were washed and IgG binding detected with horseradish peroxidase (HRP)-conjugated rabbit anti-human IgG. Substrate was developed with 3,3',5,5' tetramethylbenzidine (TMB) and absorbance was read at 450 nm. The cut-off for seropositivity was calculated as follows;

$${\text{Cut - off = (Mean + 2}} \times {\text{Standard}}\,{\text{Deviation)}}\,{\text{of}}\,{\text{pre - pandemic}}\,{\text{plasma}}\,{\text{OD}}\,{\text{values}}$$

### SARS-CoV-2 neutralizing antibody (NAbs) levels

To quantify neutralizing antibodies to SARS-CoV-2 virus in plasma, the WANTAI SARS-CoV-2 NAbs ELISA (Beijing Wantai Biological Pharmacy Enterprise; Beijing, China) was used according to manufacturer’s instructions. In summary, 100 μL of plasma and controls were added to SARS-CoV-2 spike pre-coated plates in duplicates and incubated for 60 min at 37 °C. After incubation, the plates were washed 5 × with supplied wash buffer. Following which, 100 μL of HRP-conjugated spike-RBD antibody was added. The plates were then incubated for 30 min at 37 °C and washed again 5x. Subsequently, the plates were developed by the addition of 50 μL of Chromogen Solution A and 50 μL of Chromogen Solution B per well and incubated in the dark for 15 min at 37 °C. The reaction was stopped with 50 μL of stop solution per well. The absorbance of 450 nm was measured using a Multiskan FC plate reader. Samples were analyzed in duplicates and the binding inhibition rate was calculated as:

$${\text{[(Average}}\,{\text{value}}\,{\text{of}}\,{\text{standard}}\,{\text{0U/ml - Average}}\,{\text{value}}\,{\text{of}}\,{\text{specimen)}} \times {\text{100\% ]/Average}}\,{\text{value}}\,{\text{of}}\,{\text{standard}}\,{\text{0U/ml}}$$

Samples with a binding inhibition rate ≥ 50% were considered positive.

### Data analysis

Data was analysed using Graph Pad Prism Software Version 10.0.0 (Boston, Massachusetts USA). Seropositivity rates were estimated as the proportion of individuals (and 95% confidence interval) reactive to SARS-CoV-2-specific antigens based on the seropositivity cut-off. Paired t- test was used to compare matching maternal and cord IgG levels. Transplacental efficiency was estimated as the ratio of cord IgG levels: maternal IgG levels. In all analysis, *p* value < 0.05 was considered statistically significant.

### Supplementary Information

Supplementary Information.

## Data Availability

All data generated or analysed during this study are included in this published article.
