# Supplementary material for: Efficient transplacental transfer of SARS-CoV-2 antibodies between naturally exposed mothers and infants in Accra, Ghana
Source: Sci Rep. 2024 May 10;14:10772. doi: 10.1038/s41598-024-61496-3 (PMC11087586; doi:10.1038/s41598-024-61496-3)
Supplement: Supplementary file 1 — Supplementary Information. [file 41598_2024_61496_MOESM1_ESM.docx]

Supplementary Table 1: Birth outcomes of study participants at delivery

| **Birth Oucomes, mean ± SD** | **Seronegative** | **Seropositive** | **p value** |
| --- | --- | --- | --- |
| Birth weight (kg) | 3.19 ± 0.49 | 3.17 ± 0.3 | 0.789 |
| Head circumference (cm) | 34.50 ± 1.30 | 34.57 ± 1.12 | 0.6935 |
| Length of baby (cm) | 49.48 ±3.29 | 49.00 ± 3.63 | 0.3562 |
